# Supplementary material for: Causal interplay between lactose intolerance and gut microbiota: a combined bidirectional Mendelian randomization and in vivo validation study
Source: Front Nutr. 2026 Jun 1;13:1803337. doi: 10.3389/fnut.2026.1803337 (PMC13265576; doi:10.3389/fnut.2026.1803337)
Supplement: Supplementary file 1 [file Data_Sheet_1.zip › supplementary materials/Forward/leave one out cross validation/ebi-a-GCST90027554.finngen_R12_E4_LACTONAS.pdf]

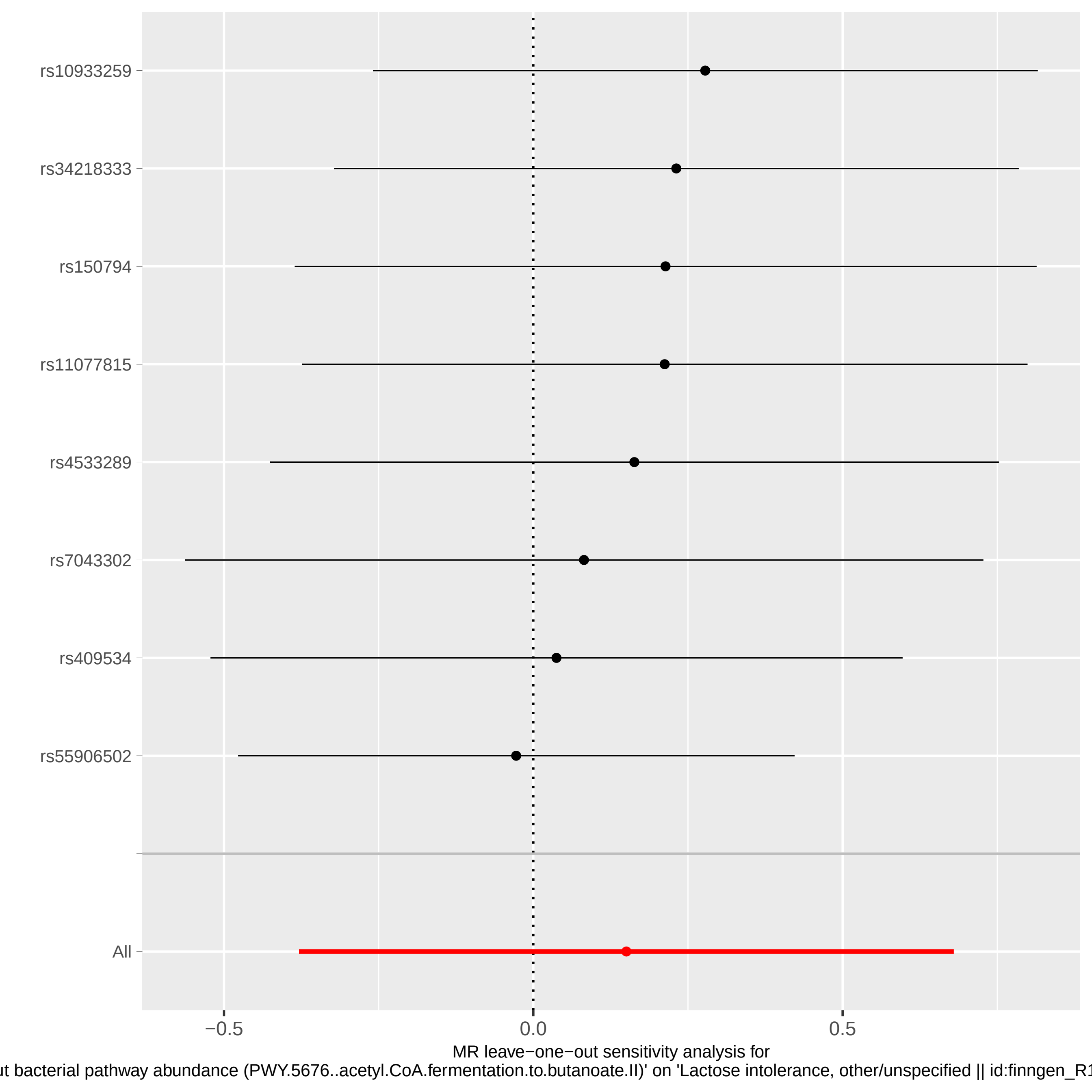

rs10933259
rs34218333
rs150794
rs11077815
rs4533289
rs7043302
rs409534
rs55906502
All
−0.5
0.0
0.5
MR leave−one−out sensitivity analysis for
ut bacterial pathway abundance (PWY.5676..acetyl.CoA.fermentation.to.butanoate.II)' on 'Lactose intolerance, other/unspecified || id:finngen_R1
